# Percutaneous transluminal angioplasty for symptomatic hepatic vein-type Budd-Chiari syndrome: feasibility and long-term outcomes

**DOI:** 10.1038/s41598-022-16818-8

**Published:** 2022-08-18

**Authors:** Aboelyazid Elkilany, Mohamed Alwarraky, Timm Denecke, Dominik Geisel

**Affiliations:** 1 Department of Diagnostic and Interventional Radiology, Charité-Universitätsmedizin Berlin, Corporate Member of Freie Universität Berlin, Humboldt-Universität Zu Berlin, Berlin Institute of Health, Augustenburger Platz 1, 13353 Berlin, Germany; 2 Department of Diagnostic Medical Imaging and Interventional Radiology, National Liver Institute, Menoufia University, Menoufia, Egypt; 3 Department of Diagnostic and Interventional Radiology, Leipzig University Hospital, Leipzig, Germany

**Keywords:** Diseases, Gastroenterology

## Abstract

For management of Budd-Chiari syndrome (BCS), a step-wise therapeutic approach starting with medical treatment, followed by endovascular recanalization, transjugular intrahepatic portosystemic shunt, and finally liver transplantation has been adopted. We retrospectively analyzed 51 patients with symptomatic short segment (≤ 30 mm) hepatic vein (HV)-type BCS who underwent percutaneous transluminal balloon angioplasty (PTBA) with/without stenting to determine the feasibility, clinical effectiveness, and long-term outcomes. The intervention was technically successful in 94.1% of cases (48/51)—32 patients underwent PTBA and 16 patients underwent HV stenting. Procedure-related complications occurred in 14 patients (29.1%). The clinical success rate at 4 weeks was 91.7% (44/48). Nine patients underwent reintervention, six patients due to restenosis/occlusion and three patients with clinical failure. The mean primary patency duration was 64.6 ± 19.9 months (CI, 58.5–70.8; range, 1.2–81.7 months). The cumulative 1-, 2-, and 5-year primary patency rates were 85.4, 74.5, and 58.3%, respectively. The cumulative 1-, 2-, and 5-year secondary patency rates were 93.8, 87.2, and 75%, respectively. The cumulative 1-, 2-, and 5-year survival rates were 97.9, 91.5, and 50%, respectively. Percutaneous transluminal angioplasty with and without stenting is effective and achieves excellent long-term patency and survival rates in patients with symptomatic HV-type BCS. With its lower incidence of re-occlusion and higher clinical success rate, HV angioplasty combined with stenting should be the preferred option especially in patients with segmental HV-type BCS.

## Introduction

Budd–Chiari syndrome (BCS) is a rare disorder defined as partial or complete obstruction of the hepatic venous outflow tract anywhere along its course from the small hepatic veins (HVs) to the suprahepatic portion of the inferior vena cava (IVC) in the absence of cardiac or pericardial disease or hepatic veno-occlusive disease/sinusoidal obstruction syndrome^1–3^.

Primary BCS is diagnosed when venous outflow is obstructed from within the vein (venous thrombosis or membranous obstruction) whereas secondary BCS is caused by compression or invasion from the outside (by tumor, abscess, cyst, etc.)^4,5^.

Based on the site of hepatic venous outflow compromise—HVs, IVC, or IVC and HVs—HV-type, IVC-type, and combined-type BCS can be distinguished^6,7^.

A stepwise therapeutic approach proceeding according to the response to therapy has been adopted starting with medical treatment (anticoagulants and diuretics), followed by percutaneous recanalization (thrombolysis, percutaneous transluminal balloon angioplasty (PTBA) with or without stenting), transjugular intrahepatic portosystemic shunt (TIPS), and finally liver transplantation (LTx) in patients who have not responded to any of the previous treatment steps^1,6,8–10^.

Recanalization of the IVC is the most commonly performed treatment in IVC-type BCS and most patients with combined-type BCS, of whom approximately 86–89% have a compensatory patent accessory HV (AHV)^6,11^.

In patients with HV-type BCS, endovascular recanalization (PTBA with/without stenting) is considered the first-line treatment specifically in patients with short segment stenosis, followed by TIPS^5,7,12,13^. TIPS is considered the first-line treatment in patients with diffuse thrombosis of HVs, where percutaneous angioplasty is not technically feasible and has low long‐term patency rates^5,14^.

The aim of the present study is to evaluate the feasibility, clinical effectiveness, and long-term outcomes of PTBA with and without stenting for endovascular treatment of HV-type BCS.

## Results

### Patient demographics

The study included 51 patients (31 female, mean age, 27.2 ± 9.1 years; range, 14–52 years). No risk factor was identified in 11 patients (21.6%) while factor V Leiden mutation (FVLM) and protein C deficiency were the most frequent risk factors, identified in 9 (17.6%) and 8 patients (15.7%), respectively. Thirty-four patients (66.7%) had occlusion of all 3 main HVs while 17 patients (33.3%) had occlusion of two HVs. Obstruction was segmental in 37 patients (72.5%; mean length of obstructed segment, 24.68 ± 4.86 mm; range, 14–30 mm). Ascites was the most common symptom (47 patients, 92.2%). A Rotterdam score of 1.22 ± 0.62 (class II), Clichy score of 5.36 ± 0.88, and revised Clichy score of 4.49 ± 1.30 suggested a moderate prognosis. Patient demographics are presented in Table 1.

**Table 1:** Patient demographics in the total study population versus subgroups managed with hepatic vein angioplasty alone and those managed with additional HV stent.

	Overall patients (n = 51)		PTBA group (n = 32)		HV stent group (n = 16)		*P*-value
	N (%)	Mean ± SD (min − max)	N (%)	Mean ± SD	N (%)	Mean ± SD	
Age (years)	51	27.2 ± 9.1 (14 – 52)	32	26.8 ± 9	16	26.9 ± 9.1	0.991
Sex (female/male)	31/20 (60.8/39.2%)		22/10		8/8		0.206
Duration between first symptom and treatment (months)	51	6.48 ± 3.02 (1.8—12.4)	32	6.08 ± 2.7	16	6.35 ± 3.2	0.757
**Risk factors**							0.802
No risk factor identified	11 (21.6)		7		3		
Factor V leiden mutation (FVLM)	9 (17.6)		5		3		
Methylene tetrahydrofolate reductase (MTHFR) mutation	6 (11.8)		2		3		
Myeloproliferative disorder (MPD)	2 (3.9)		2		0		
Anti-phospholipid syndrome	5 (9.8)		4		1		
Anti-thrombin III deficiency	4 (7.8)		2		2		
Protein C deficiency	8 (15.7)		5		3		
Protein S deficiency	5 (9.8)		4		1		
Systemic lupus erythematosus (SLE)	1 (2.0)		1		0		
**Clinical presentation**							
Ascites	47 (92.2)		31 (96.95)		13 (81.35)		0.101
Abdominal wall collaterals	10 (19.6)		7 (21.9%)		2 (12.5%)		0.697
Esophageal/fundal varices	8 (15.7)		4 (12.5%)		3 (18.8%)		0.672
Jaundice	12 (25)		8 (25%)		4 (25%)		1.000
Liver cirrhosis	9 (17.6)		3 (9.4%)		3 18.8%)		0.386
Abdominal pain	36 (70.6)		22 (68.8%)		12 (75%)		0.746
Abdominal distension	36 (70.6)		22 (68.8%)		13 (81.35)		0.497
Hepatomegaly	39 (76.5)		24 (75%)		14 (87.5%)		0.460
Hepatic encephalopathy	10 (19.6)		4 (12.5%)		4 (25%)		0.413
Lower limb edema	10 (19.6)		5 (15.6%)		3 (18.8%)		1.000
**Prognostic indices**							
Child–Pugh score	51	8.65 ± 1.45	32	8.50 ± 1.566	16	9.00 ± 1.211	0.269
Child–Pugh class (A/B/C)	5/32/14 (9.8/62.7/27.5%)		5/18/9 (15.6/56.3/28.1%)		0/12/4 (0/75/25%)		0.208
MELD score	51	13.4 ± 2.6 (8–19)	32	13.13 ± 2.89	16	13.8 ± 2.01	0.399
Clichy score	51	5.36 ± 0.88 (3.49–6.98)	32	5.298 ± 0.923	16	5.440 ± 0.875	0.613
Revised Clichy score	51	4.49 ± 1.30 (1.79–8.16)	32	4.281 ± 1.265	16	4.802 ± 1.275	0.186
Rotterdam score	51	1.22 ± 0.62(0.008–2.32)	32	1.174 ± 0.477	16	1.171 ± 0.794	0.988
Rotterdam class (class 1/3)	41/10 (80.4/19.6%)		28/4 (87.5/12.5%)		12/4 (75/25%)		0.413
Nature of obstruction (membranous/Segmental)	14/37(27.5/72.5%)		10/22 (31.3–68.8%)		4/12 (25–75 5)		0.746
Mean length of occluded HV segment (mm)	37	24.68 ± 4.86 (14–30)	22	22.73 ± 5.101	12	27.33 ± 2.871	**0.002**
**Occluded HV**							
Right + Middle + Left HVs	34 (66.7%)		23		8		0.147
Right + Middle HVs	6 (11.8%)		2		4		
Left + Middle HVs	11 (21.6%)		7		4		
**Treatment approach**							
Transjugular/Combined transjugular-transhepatic	33/18 (64.7/35.3%)		22/10		11/5		1.000
**Number of recanalized target HVs**							
One HV	43 (89.6%)		27		16		0.118**
Two HVs	5 (10.4%)		5		0		
**Target vein for recanalization:**							
Right HV	25 (52.1%)		15		10		0.223
Middle HV	18 (37.5%)		12		6		
Right + Middle HVs	5 (10.4%)		5		0		
Inflation time (min)	48		32	7.50 ± 2.185	16	8.88 ± 1.455	**0.027**
**HV recoil (15-min recoil test):**							
No/yes*	32 (66.7%)/16 (33.3%)	7.96 ± 2.06 (4–12)	32/0		0/16		–
FHVP before angioplasty (cmH_**2**_**O)**	48	43.13 ± 6.64 (33–57)	32	43.88 ± 5.94	16	41.63 ± 7.84	0.273
FHVP after angioplasty (cmH_**2**_**O)**	48	15.35 ± 2.20 (11–19)	32	15.03 ± 2.19	16	16.00 ± 2.13	0.152
Primary patency duration (months)	48	33.68 ± 19.9 (1.2–81.7)	32	32.25 ± 21.30	16	36.53 ± 17.02	0.489
Secondary patency duration (months)	44	34.31 ± 19.05	28	34.38 ± 20.71	16	34.05 ± 18.17	0.984
Overall survival duration (months)	48	40.11 ± 6.83	32	39.78 ± 17.38	16	40.78 ± 16.21	0.847
Death	2		1		1		0.482
Clinical success at 2 weeks (No/partial/complete)	3/27/18		3/18/11 (9.4/56.3/34.3%)		0/9/7 (0/56.3/43.8%)		0.417
Clinical success at 1 month (No/partial/complete)	4/9/35		4/4/24 (12.5/12.5/75%)		0/5/11 (0/31.3/68.8%)		0.132
Reintervention (Stent/TIPS)	5/4 (55.6/44.4%)		3/4 (42.9/57.1%)		2/0		0.444
**Complications**							
HV thrombosis (thrombosis/restenosis) ***	6 (12.5%)***		2/2 (6.3–6.3%)		2/0 (12.5%–0)		0.472
Refractory ascites	2 (4.2%)		2 (6.3%)		0		0.546
Refractory collaterals	3 (6.3%)		3 (9.4%)		0		0.541
Pulmonary embolism	3 (6.3%)		2 (6.3%)		1 (6.3%)		1.000
HV dissection	1 (2.1%)		0		1 (6.3%)		0.333
Intraperitoneal bleeding	2 (4.2%)		2 (6.3%)		0		0.546
Intrahepatic hematoma	2 (4.2%)		2 (6.3%)		0		0.546
**Laboratory values**							
Total bilirubin	51	2.05 ± 0.45 (1.12–2.93)	32	1.97 ± 0.50	16	2.19 ± 0.33	0.116
Albumin	51	3.02 ± 0.48 (2.10–4.20)	32	3.09 ± 0.51	16	2.89 ± 0.38	0.181
AST	51	150.82 ± 76.78 (39–373)	32	137.81 ± 76.08	16	168.94 ± 79.04	0.194
ALT	51	171.16 ± 87.02 (25–352)	32	151.47 ± 83.54	16	200.06 ± 90.10	0.071
ALP	51	129.88 ± 66.44 (29–411)	32	121.66 ± 71.64	16	141.63 ± 58.87	0.341
INR	51	1.40 ± 0.28 (0.90–2.10)	32	1.43 ± 0.28	16	1.39 ± 0.27	0.762
PT	51	17.52 ± 3.46 (11.25–26.25)	32	17.81 ± 3.56	16	17.48 ± 3.43	0.762
Creatinine	51	0.96 ± 0.24 (0.52–1.54)	32	0.91 ± 0.24	16	1.04 ± 0.19	0.052

*MELD* model for end-stage liver disease, *HV* hepatic vein, *FHVP* free HV pressure, *AST* aspartate aminotransferase, *ALT* alanine aminotransferase, *ALP* alkaline phosphatase, *INR* international normalized ratio, *PT* prothrombin time.

*Including 2 patients (4.2%) with membranous obstruction in whom HV restenosis occurred.

**Fisher’s exact test.

***Including 4 patients (8.3%) with membranous obstruction with immediate recoil following dilatation and 12 patients with segmental obstruction (25%) in whom HV re-thrombosis occurred.

### Technical success

Endovascular recanalization was technically successful in 48 patients (94.1%). Thirty-two patients underwent PTBA alone (62.7%) and 16 patients (31.4%) underwent PTBA with HV stent insertion. Angioplasty was not successful in 3 patients (5.9%) with occlusion of all 3 Main HVs. These patients were treated with TIPS insertion.

**Figure 1:**
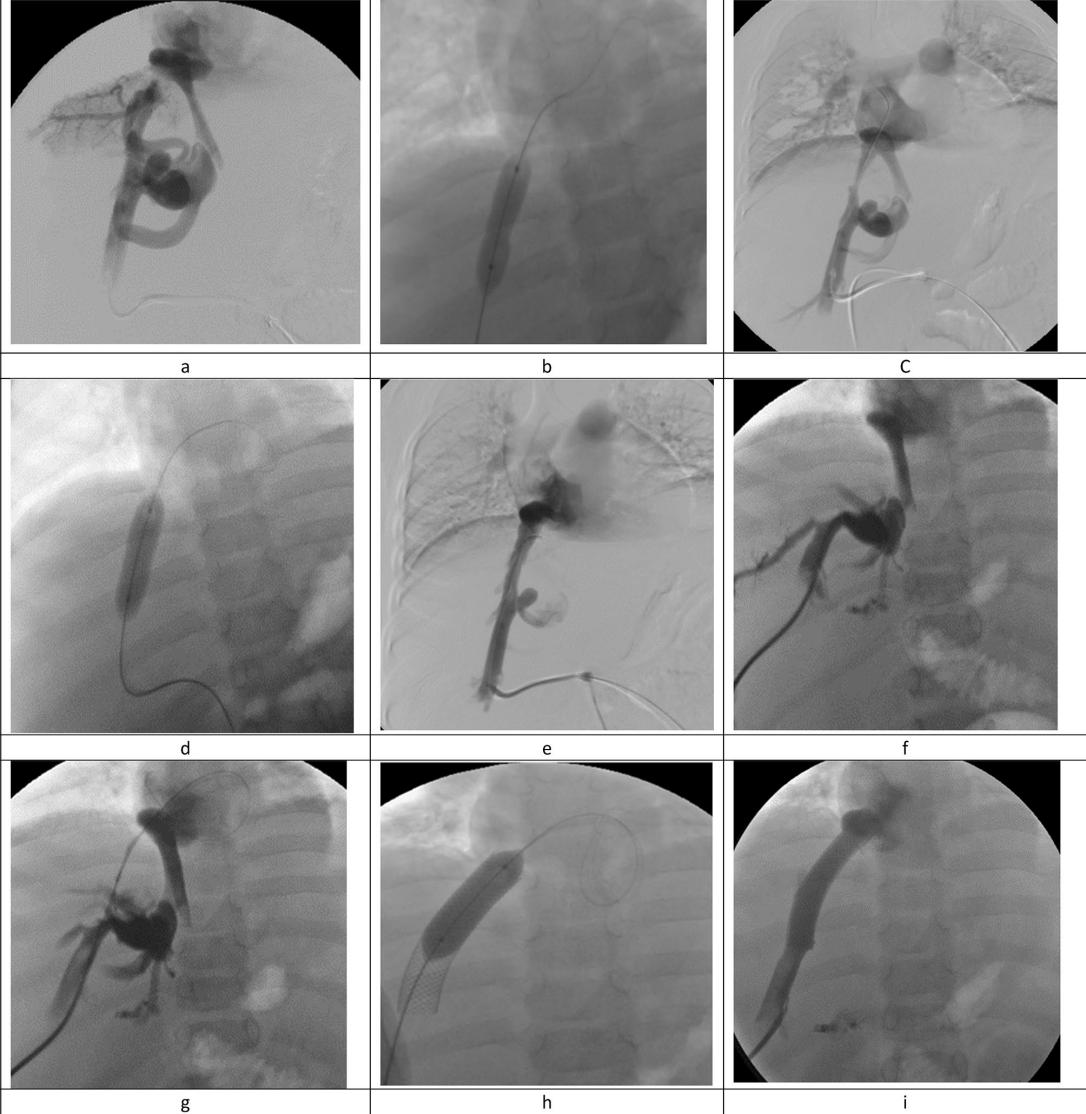
Angiography images of a 15-year-old female patients with FVLM: factor V Leiden mutation (FVLM) with obstruction of the right hepatic vein for 6 months. Transhepatic venography revealed short segment obstruction involving right HV ostium with multiple dilated vascular collaterals (**a**). Balloon dilatation was performed (**b**), venography revealed residual stenosis and persistence of collaterals (**c**). Repeat dilatation was performed (**d**) and control venography revealed good recanalization of the HV with disappearance of vascular collaterals and without residual stenosis (**e**). Patient presented with recurrent symptoms 10 months following HV angioplasty, and color Doppler ultrasonography revealed thrombosis of the previously treated right HV. Angiography revealed progressive thrombosis of the treated HV compared to the primary intervention with vascular collaterals (**f**). The obstruction was bypassed using a transhepatic approach (**g**) followed by stent insertion (**h**). Final venography following stent dilatation revealed free flow of contrast agent across the stent with disappearance of intrahepatic collaterals (**j**).

Recanalization of one main HV was sufficient to restore hepatic venous drainage in most of the patients (43/48), while recanalization of 2 HVs was necessary in 5 patients (10.4%). The right HV was most frequently selected as the target vein in 25 patients (52.1%, Fig. 1). Mean free HV pressure (FHVP) significantly decreased from 43.13 ± 6.64 before recanalization to 15.35 ± 2.20 after treatment (*P* value < 0.001). Descriptive analysis of interventional details is listed in Table 1.

### Procedure-related complications

Procedure-related complications were found in 14 patients (29.1%). Intraperitoneal bleeding and subcapsular hematoma occurred in 2 patients managed with a combined transhepatic-transjugular approach. Subsegmental pulmonary embolism was observed during balloon angioplasty in 3 patients who suffered a drop in O_2_ saturation. The diagnosis was confirmed by postinterventional pulmonary computed tomography angiography (CTA). No stent migration was noted after HV stenting. All complications were successfully managed by conservative treatment. All complications were minor (classes A and B according to North American Society of Interventional Radiology (SIR) classification)^15^.

### Clinical success

The clinical success rate was 93.7% (45/48) at two weeks and 91.7% (44/48) at four weeks. In patients with clinical success, BCS-related symptoms were significantly relieved within one month of the intervention (complete success rate of 72.9% (35/44 patients) vs. 37.5% (18/45 patients) two weeks following angioplasty). There was no clinical improvement in 3 patients at two-week follow-up despite target HV patency in follow-up Doppler US and venography. Clinical failure in these patients was attributed to deterioration of liver function since they had a long history of BCS complicated by cirrhosis. Those patients were managed by TIPS shunt insertion. In addition, improvement in dilated vascular collaterals (abdominal wall collaterals or varices) was observed earlier than relief of other presenting symptom such as abdominal distention and ascites, which diminished gradually over a longer period of time.

Mean serum total bilirubin improved from 2.05 ± 0.45 mg/dl (range 1.12–2.93) before treatment to 1.2 ± 0.31 mg/dl (0.72–2.10) two weeks after treatment (*P* = 0.01). Similarly, serum total bilirubin, albumin, aspartate aminotransferase, and alanine aminotransaminase levels significantly improved one month after the intervention (*P* value < 0.001).

### Patency

**Figure 2:**
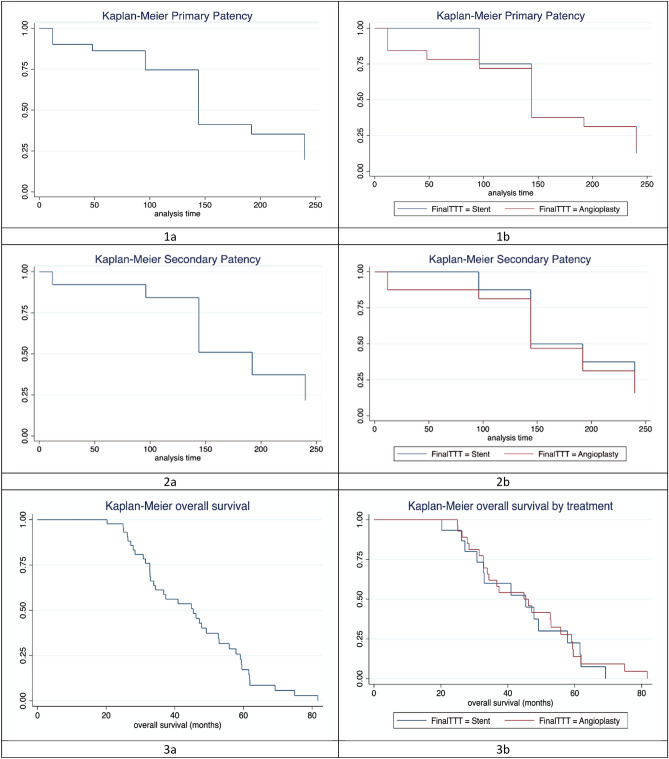
Kaplan–Meier analysis of primary patency (**1a**, **b**), secondary patency (**2a**, **b**), and survival time (**3a**, **3b**) in the total study population (**1a**, **2a**, **3a**) and subgroup analysis according to type of intervention (**1b**, **2b**, **3b**).

The mean follow-up duration was 40.1 ± 16.8 months (95% CI 35.2–45 months, range 8.8–81.7 months). Eight patients were lost to follow-up. Re-obstruction was in the form of re-thrombosis of the target HV in 4 patients with segmental HV thrombosis and restenosis/occlusion in 2 patients with membranous obstruction. The mean primary patency duration was 64.6 ± 19.9 months (95% CI, 58.5–70.8 months; range, 1.2–81.67 months). The cumulative 1-, 2-, 3-, and 5-year primary patency rates were 85.4, 74.5, 65.5, and 58.3%, respectively (Table 2, Fig. 2).

**Table 2:** Descriptive analysis of primary and secondary patency rates at different time points over 5 years. CI, confidence interval.

Follow-up	Overall number	Primary patency rate			Secondary patency rate			Overall survival rate		
		N	%	95% CI	N	%	95% CI	N	%	95% CI
1 year	48	41	85.4	72.2–93.9	45	93.8	82.8–98.7	47	97.9	88.9–99.9
2 years	47	35	74.5	59.7–86.1	41	87.2	74.3–95.2	43	91.5	79.6–97.6
3 years	29	19	65.5	45.7–82.1	25	86.2	68.3–96.1	25	80.6	62.5–92.5
4 years	22	15	68.2	45.1–86.1	17	77.3	54.6–92.2	17	68	46.5–85.1
5 years	12	7	58.3	27.2–84.8	9	75	42.8–94.5	9	50	26–74

### Reintervention

Nine patients underwent reintervention—six patients due to restenosis/occlusion of the target HV and 3 patients with clinical failure manifesting as refractory ascites/vascular collaterals despite patent recanalized target HV. Among the 9 patients, 5 underwent HV stent insertion (3 patients who were primarily managed with PTBA and 2 patients primarily managed with HV stent, *P* = 0.44) while 4 patients underwent TIPS insertion (one patient with segmental HV re-thrombosis following PTBA and 3 patients in whom clinical failure was encountered despite a patent recanalized target vein).

Among the 9 patients, 5 underwent a short-term reintervention after a primary patency duration of 1.2–2.43 months (4 patients underwent TIPS insertion and one patient who was primarily managed with PTBA underwent HV stent insertion).

The mean secondary patency duration was 64.2 ± 2.7 months (95% CI, 58.9–69.6 months; range, 12.1–81.7 months). The cumulative 1-, 2-, 3-, and 5-year secondary patency rates were 93.8, 87.2, 86.2 and 75%, respectively (Table 2, Fig. 2).

In ROC analysis of different prognostic indices for prediction of target vein patency following intervention, the Clichy score and Rotterdam score demonstrated the largest AUC of 0.798 for prediction of patency (CI of 0.000–0.464 and 0.255–0.379 for Clichy and Rotterdam scores, respectively) (Table 3).

**Table 3:** Receiver operator characteristics of different prognostic indices as predictors of primary patency.

	N of features	ROC area	SE	[95% conf. interval]	
Clichy score	48	0.7976	0.1334	0.0000	0.46387
Revised Clichy score	48	0.6667	0.1257	0.08705	0.57962
Rotterdam score	48	0.7976	0.0903	0.02546	0.37930

**Figure 3:**
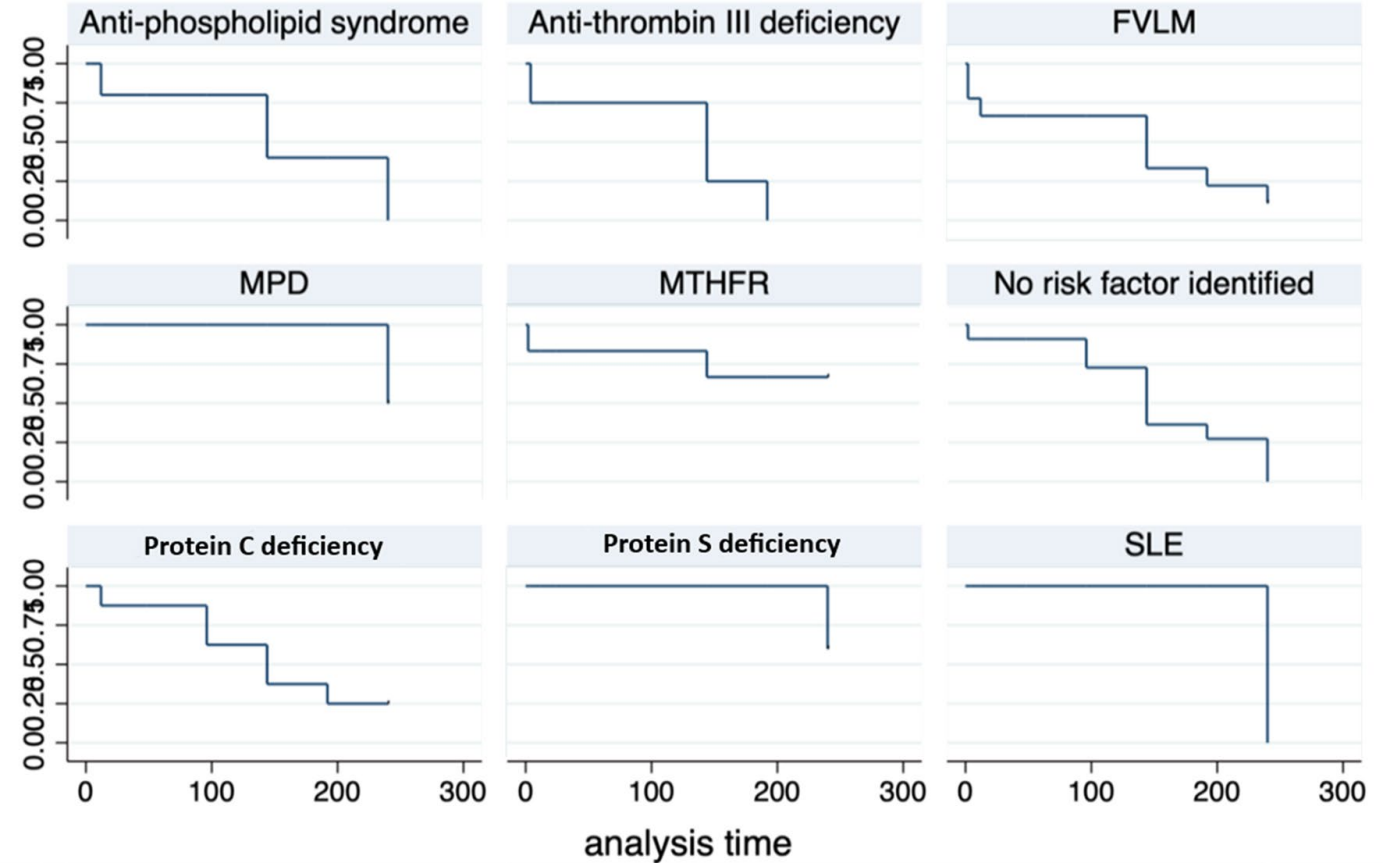
Regression analysis of different risk factors for development of Budd Chiari syndrome for prediction of hepatic vein patency following recanalization. MPD, myeloproliferative disorder; MTHFR, methylene tetrahydrofolate reductase; FVLM, factor V Leiden mutation; SLE, systemic lupus erythematosus.

In univariate regression analysis, no significant factors influencing HV patency following angioplasty or patient survival either in the whole study population or in subgroup analysis of patients with segmental obstruction were identified (Fig. 3, Supplementary Tables 1, 2, 3 and 4).

### Survival

The mean survival time was 50.62 ± 5.33 months (95% CI 40.17–61.08). The cumulative 1-, 2-, 3-, and 5-year survival rates were 97.9, 91.5, 80.6, and 50%, respectively (Fig. 2). Two patients died 13.4 and 8.8 months after treatment. Causes of death were liver failure with variceal bleeding (n = 1) and fulminant liver failure related to hepatitis B (n = 1).

### Comparison of PTBA alone and PTBA with HV stent

There was no significant difference in the re-obstruction rate between patients who were managed with PTBA alone (n = 4/32, 12.5%) and those who underwent additional HV stent insertion (n = 2/16, 12.5%, *P* = 0.472). The occluded segment in the HV stent group was significantly longer than in the PTBA group (27.3 mm vs. 22.7 mm, *P* = 0.002). The complete clinical success rate 2 weeks following the intervention was higher in the HV stent group (7/16 patients, 43.8%) than in the PTBA group (11/32, 34.3%, *P* = 0.42). No significant difference in complications was noted. Refractory ascites and refractory vascular collaterals were only observed in the PTBA group (2 and 3 patients, *P* = 0.55 and 0.54, respectively). Two patients experienced PE in the PTBA group vs. one in the HV stent group (*P* = 1.000).

In subgroup analysis of patients in whom angioplasty was not technically successful (n = 3, managed by TIPS) and patients in whom PTBA with or without stenting was complicated by either re-occlusion (n = 6) or clinical failure (n = 3), we observed that (a) the duration of BCS-related symptoms before the intervention was significantly longer (9.8 ± 2.1 vs. 5.4 ± 2.5 months, *P* = 0.000) and that (b) FHVP before the intervention was significantly higher (47.1 ± 6.7 vs. 42.2 ± 6.4 cmH_2_O, *P* = 0.04). Ten patients had segmental HV obstruction while only two patients had membranous obstruction. The length of the occluded segment was longer in these patients (26.1 ± 4.4 mm vs. 24.2 ± 5 mm, *P* = 0.284). Furthermore, eleven patients had occlusion of all three Main HVs, and one patient had occlusion of two HVs. In the nine patients who underwent reintervention, 7 patients were primarily managed with PTBA while only two patients had HV stent placement.

## Discussion

For management of BCS, a step-wise treatment strategy starting with medical treatment, followed by endovascular revascularization and TIPS shunt insertion and finally LTx has proved to be effective and achieves good long-term survival^7,16^.

The primary aim of HV recanalization is to relieve liver congestion, improve liver functions, and alleviate patients’ symptoms^11,13,17^. Recently, HV recanalization is being increasingly recognized and recommended in the EASL and APASL guidelines for BCS as the most preferred invasive radiological intervention in patients with BCS, especially in patients with short segment HV thrombosis or ostial stenosis since it restores hepatic circulation closest to physiology^1,18^.

The effectiveness of medical treatment is controversial. Earlier studies reported medical treatment alone to be ineffective and associated with poor long-term outcome^17,19,20^. On the other hand, Kulkarni et al.^8^ and Zeitoun et al.^21^ observed no significant survival benefit of percutaneous recanalization or surgical shunting compared to patients managed by medical treatment alone. In patients with persistent symptoms, endovascular intervention (PTBA with or without stenting) is performed, which is most effective in patients with short segment HV or IVC obstruction^6,12,13,17,22,23^, whereas TIPS is reserved for symptomatic patients in whom endovascular management has failed or is not technically feasible such as patients with diffuse HV thrombosis or combined-type BCS^11,24–27^. Indications for LTx include end-stage chronic liver disease due to progressive deterioration of liver function despite medical and/or interventional management (10–20% of BCS patients), fulminant liver failure as well as selected patients with BCS complicated by hepatocellular carcinoma (HCC) and still eligible for LTx^5,28,29^. The present study investigated technical success, clinical effectiveness, and long-term outcomes of endovascular treatment in HV-type BCS.

The etiology of BCS is known to be variable with thrombosis being more common in western countries and membranous obstruction in the Asian population^30,31^. The results of our study are consistent with previous findings obtained in patients with BCS in Egypt with FVLM, protein C deficiency, and methylene tetrahydrofolate reductase (MTHFR) mutation as the most common prothrombotic risk factors in Egyptian BCS patients^32–34^.

The good technical and clinical success achieved in the present study further corroborates the strategy recommended by several previous study groups, namely that recanalization of one HV with the shortest obstruction should be sufficient to drain the entire liver parenchyma and relieve hepatic venous flow compromise because of the well-established intrahepatic collateral circulation in patients with BCS^12,13,17,22,35,36^.

The primary patency rates and patency duration in our patients are comparable to several previous studies investigating endovascular treatment in HV-type BCS^6,12,13,17,22,36–39^. In addition, the high cumulative secondary patency rates suggest that endovascular HV recanalization is repeatable when restenosis occurs^17^. These good long‐term patency and survival rates further corroborate the stepwise approach in the management of BCS. In a meta-analysis by Zhang et al. including over 2000 BSC patients, percutaneous HV recanalization was found to have a technical success rate of 93.1% (CI 91.8–94.3) and 1-year and 5-year survival rates of 95.9% (CI 93.4–98.3) and 88.6% (CI 82.4–94.8), respectively^40^.

The primary and secondary 5-year patency rates in the present study were significantly lower than in the study of Ding et al. (90 and 98.6%, respectively)^17^. This could be explained by the larger diameter of balloons (12–20 mm vs. 14 mm in our study) as well as the significantly larger proportion of patients with membranous obstruction (91 patients vs. 2 patients with segmental obstruction) in their study.

In the present study, HV recanalization was primarily performed using PTBA alone with HV stenting being reserved for cases with residual stenosis/restenosis or recurrent/refractory symptoms following PTBA. Although statistically not significant, the reintervention rates due to re-occlusion/re-stenosis or clinical failure were higher in patients who underwent PTBA alone than those who were managed by HV stent placement, especially for segmental obstruction. This observation suggests that PTBA combined with stent insertion might be superior to PTBA alone in endovascular recanalization of HV-type BCS, increasing the clinical success rate and lowering the frequency of re-occlusion. The nonsignificant difference might be explained by the relatively small number of patients in our study. Similarly, Eapen et al. suggested that re-occlusion was more common in patients undergoing PTBA alone than in patients with additional stent placement; however, the difference was not statistically significant^41^. In the study by Han et al., PTBA alone without stenting was a predictor of re-occlusion, which was in turn a predictor of survival in univariate and multivariate regression analysis. Consequently, they recommend PTBA and stenting to lower the incidence of reintervention and improve survival^6^. Conversely, Cheng et al. recommend to reserve HV stenting for patients with residual stenosis > 25% and a pressure gradient across the stenosis/occlusion > 3 mmHg, while, in general, PTBA should be the preferred option since stent implantation is permanent and might increase the risk of complications and render reintervention in case of occlusion more difficult^10^. We exclusively used uncovered stents for HV recanalization. Cheng et al. suggest that uncovered stents are superior since covered stents might hinder development of collateral circulation, which in turn could affect the clinical response following angioplasty^10^.

The majority of patients, either with technical failure or those who underwent reintervention, had segmental HV obstruction, and the length of the occluded segment was nonsignificantly longer in these patients. However, segmental HV obstruction was not an independent risk factor for re-occlusion in our analysis, contrary to previous studies by Cui et al.^13^ and Chen et al.^7^ This could be explained by the fact that we only included patients with short segment stenosis/occlusion < 30 mm.

Neither age nor sex had an effect on patency following recanalization or patient survival in our univariate analysis, which is consistent with previous studies by Sakr et al.^33^ and Qi et al.^42^. None of the prognostic indices we analyzed turned out to be a significant predictor of primary patency or survival in our analysis, and this might be explained by the low number of patients as well as high survival and patency rates. Our results are in agreement with previous studies by Chen et al.^7^ and Cui et al.^13^ but disagree with several other studies. In the study by Sakr et al., none of the prognostic indices was a significant predictor of one-year patency while the revised Clichy score was an independent predictor of one-year survival with a cut-off of 3.75^33^. In the study by Rautou et al., all prognostic indices, except the BCS-TIPS score, were significant predictors of transplant-free and invasive therapy-free survival^43^. In the study by Han et al., the Child–Pugh score, Clichy score, and BCS-TIPS score were significant predictors of survival in univariate analysis^6^. In the study by Tripathi et al., the Child–Pugh score, Model of End Stage Liver Disease (MELD) score, revised Clichy score, and Rotterdam score were significant predictors of survival in univariate analysis in addition to age^22^. However, the use of prognostic indices has so far been limited to the stratification of patients in clinical studies. Several reports suggest that prognostic indices are of limited use in individual patient management and should not be relied on for decision making about the type or timing of interventions^22,33,42,43^.

Recanalization was not complicated by hepatic encephalopathy (HE) in the present study. The incidence of HE following venoplasty is minimal because the normal physiologic pathway of hepatic blood flow is restored compared to TIPS, where portal flow is diverted, which is associated with a 17% risk of HE and deterioration of liver function by decreasing hepatopedal portal flow in segmental branches^17,39,44^. Tripathi et al. investigated venoplasty in 63 patients with BCS and compared their results with a previously reported series of 59 patients treated by TIPSS. Angioplasty yielded similar patency and survival rates while it is less invasive and has significantly fewer procedure-related complications (9.5% vs. 27.1%) and HE (0% vs. 18%) in comparison to TIPS^22^.

The good patency and survival rates in the present study suggest that HV angioplasty should be preferred over medical treatment even in patients with early HV-type BCS (i.e., without symptoms of portal hypertension) to prevent deterioration of liver function and development of portal hypertension-related symptoms. Kulkarni et al.^8^ reported that, despite a nonsignificant difference in terms of patient survival, patients treated by percutaneous recanalization had significantly lower rates of recurrent symptoms and hospital admissions compared with medical treatment, which is in agreement with our results. Furthermore, Shin et al. report success rates of 33–54% and 0–7% with medical management alone in patients with early HV-type and combined-type BCS, respectively^45^. These rates are relatively low compared to endovascular interventions.

Anticoagulant therapy was initiated during the intervention and continued afterwards as suggested by Zhang et al. They strongly recommended anticoagulant therapy following stent insertion in patient with segmental HV and/or IVC obstruction since patients without anticoagulant therapy following venoplasty had a higher incidence of stent occlusion^46^.

### Limitations

This study has some limitations. First, the retrospective study design could be a source of selection bias. Second, a major limitation in the present study was measurement of the FHVP as an indicator of technical success following the intervention without measuring the wedged hepatic venous pressure (WHVP) and consequently hepatic vein pressure gradient (HVPG) which would have been a more accurate indicator of technical success. Currently, measurement of HVPG is considered a “gold standard” to evaluate and diagnose portal hypertension and for risk stratification in patients with liver cirrhosis since it reflects the degree of architectural disruption of the liver and provides prognostic information about the degree of liver cirrhosis^47,48^. Third, the sample size was small, especially the subset of patients who underwent HV stenting. Forth, the number of patients managed with PTBA versus those with stent insertion was not the same, which might have rendered the nonrandomized, noncontrolled comparison between the two interventions less accurate. Further prospective randomized controlled studies are recommended to investigate the potential advantage of HV stenting over PTBA alone.

### Conclusion

Endovascular hepatic vein recanalization using angioplasty with or without stenting is effective in patients with HV-type BCS and achieves excellent long-term patency and survival rates. With its lower incidence of re-occlusion and higher clinical success rate, HV angioplasty combined with stenting should be the preferred option especially in patients with segmental HV-type BCS.

## Patients and methods

### Patient population and study design

**Figure 4:**
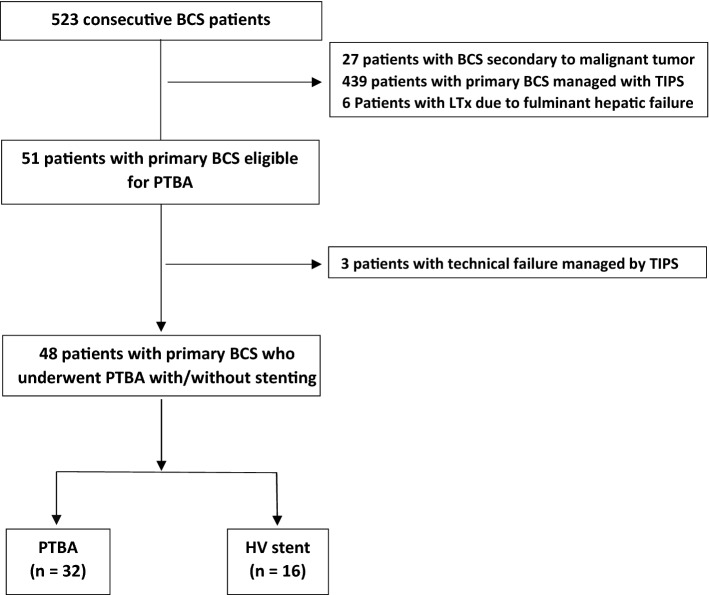
Flow chart of inclusion and exclusion of BCS patients. BSC, Budd Chiari syndrome; TIPS, transjugular intrahepatic portosystemic shunt; LTx, liver transplantation; HV, hepatic vein; PTBA, percutaneous transluminal balloon angioplasty.

We retrospectively identified 51 patients with symptomatic short segment HV-type BCS (≤ 3 cm) who underwent endovascular treatment with PTBA with or without stenting at our institution between October 2013 and January 2019 (Fig. 4) by examination of the picture archiving and communication system (PACS) and patients’ electronic medical records. The Study was performed in accordance with the relevant guidelines and regulations (Declaration of Helsinki) and approved by the institutional review board (Ethics committee—National Liver Institute). Informed consent was waived by the ethics committee ((Ethics committee—National Liver Institute).

Exclusion criteria were: long segment HV occlusion (> 3 cm), IVC-type or combined-type BCS, diffuse thrombosis of the 3 main HVs, asymptomatic BCS due to well-established intrahepatic vascular collateral, BCS secondary to malignant tumor, clinical success of medical treatment (anticoagulation and diuretics), portal vein thrombosis, and previous management with TIPS, surgical shunt, or LTx.

### Diagnosis, definition, and evaluation of symptomatic HV-type BCS

The diagnosis of BCS was established in accordance with the guidelines of the European Association for the Study of the Liver (EASL)^1^. HV-type BCS was primarily diagnosed using abdominal Doppler ultrasonography (US) and CTA or magnetic resonance angiography (MRA) when obstruction exclusively involved the HVs^4,49^. Symptomatic BCS was diagnosed when any of the following clinical manifestations was present: ascites, abdominal distention, abdominal pain, hepatomegaly, jaundice, variceal bleeding, dilated vascular collaterals of the thoraco-abdominal wall, or hepatic encephalopathy^6^.

### Laboratory parameters and severity indices for BCS

Liver function tests (LFTs) (aspartate aminotransferase [AST], alanine aminotransferase [ALT], alkaline phosphatase [ALP], serum total bilirubin, and serum albumin), serum creatinine, international normalized ratio (INR), and prothrombin time (PT), performed within three days before the intervention, were selected for the present analysis.

The Child–Pugh score^50^, MELD score^51^, and specific prognostic indices developed for BCS, including the original Clichy score^21^, revised Clichy score^52^, and Rotterdam score^53^, were calculated using the findings obtained at the time of HV recanalization. Prior to the intervention, patients with high-grade (grades III and IV) esophageal varices (n = 8) underwent prophylactic endoscopic management.

### Interventional procedures

All interventional procedures were performed under local anesthesia and conscious. Coagulation disorder was corrected aiming at INR < 2.0.

### Target vein selection

The main criterion for selection of the target HV was the length of the occlusion. From the three main HVs, the one with the shortest obstruction was selected. Other selection criteria were good caliber (≥ 7 mm), straight course, and echo-free lumen of the vein. If all three HVs were eligible according to these criteria, our preference was the right HV (easier to manipulate), followed by a common ostium of left and middle HVs, assuming that nearly 50% of the liver parenchyma can be drained by either selection. A compensatory (diameter ≥ 5 mm) but obstructed AHV would have been chosen as the target vein if the occluded segment of it was shorter than that of any of the three main HVs. However, we did not encounter any patients in whom the AHV could have been selected. Computed tomography angiography or MRA was used for primary measurement of length of obstruction and selection of the target vein. Final selection was based on HV angiography during the intervention.

### Approaches for percutaneous HV recanalization

A transjugular approach was the primary route for PTBA. All patients underwent US-guided internal jugular vein (IJV) puncture (right IJV access in 48 patients, left IJV access in 3 patients due to right IJV thrombosis). Inferior vena cava angiography was performed using a 5-Fr straight catheter with multiple side holes (Cook Medical, Bloomington, USA) to ensure patency and reveal morphology of the IVC and orifices of the HVs. A Rösch-Uchida transjugular liver access set device (Cook Medical, Bloomington, USA) was used to traverse the occluded HV segment.

A combined transjugular-transhepatic approach was used in patient with failed HV access through the jugular vein (n = 18). In this approach, percutaneous transhepatic US-guided access of the target HV was accomplished using a Neff percutaneous access set (Cook Medical) or 21-gauge Chiba needle (Cook Medical) followed by HV angiography through a 6-Fr sheath (Terumo, Tokyo, Japan). Paracentesis was performed in patients with ascites (n = 14) before the percutaneous transhepatic approach. The occlusion/stenosis was negotiated using different sets of guidewires (0.035″ angled tip and straight hydrophilic standard and stiff guidewires (Radiofocus M; Terumo) or Glidewire (Boston Scientific, Natick, Mass, USA) and catheters including a 5-Fr Cobra 2 angiographic catheter (Radiofocus, Terumo; Cordis, Warren, New Jersey, USA) and 4-Fr Headhunter catheter (Cook Medical). If the obstructed segment could not be traversed with this technique, the stiff back end of the guidewire was advanced very slowly under continuous fluoroscopic guidance and road-mapping, limiting each movement to maximum of 3 mm.

After successful passage of the occlusion/stenosis, the guidewire was snared in the IVC or right atrium using a gooseneck snare (Amplatz snare, Medtronic, Minneapolis, MN, USA) and withdrawn through the jugular sheath. Hepatic vein angiography was performed to demonstrate morphology of the HVs, length and location of the stenosis/occlusion, and presence of intrahepatic collaterals. Angioplasty with/without stenting was performed through the jugular approach as it facilitated introduction of larger-caliber balloons without increasing the risk of hepatic capsular injury compared with use of the transhepatic access.

### Percutaneous balloon angioplasty (PTBA)

Balloon angioplasty was performed using balloon catheters (Cook; Cordis; or Bard, Murray Hill, NJ, USA) of various sizes (8–14 mm diameter, 40–60 mm length). Balloons were dilated over a stiff hydrophilic (Terumo) or extra-stiff guidewire (0.035′′ Super Stiff Amplatz guidewire, J-Tip, Boston Scientific, Marlborough, MA). The balloon was manually dilated (2–11 times), with each dilatation procedure lasting approximately 1 min until the waist disappeared. Repeat HV venography was performed 15 min after dilatation (15 min recoil test) to look for significant residual stenosis, recoiling, or persistence of intrahepatic collaterals.

### Self-expandable stent placement

Metal stents of 10–14 mm diameter and 40–60 mm length (Wallstent; Boston Scientific) were inserted when initial balloon dilatation was insufficient as evidenced by any of the following criteria in HV venography after 15 min: (a) significant residual stenosis of > 30% or recoiling following angioplasty; (b) pressure gradient > 15 cmH_2_O (1 cmH_2_O = 0.098 kPa) between HV segments proximal and distal to the occlusion/stenosis; and (c) persistence of intrahepatic vascular collaterals.

The balloon or the stent extended at least 10 mm beyond the lesion ends. The diameter of the stent or balloon needed to be 2 mm larger than the diameter of the target HV. Free HV pressure was measured by a piezometer tube before and after recanalization. At the end of the procedure, the transhepatic track was embolized using gel foam strips pushed through the introducer sheath.

## Postprocedure management and follow-up

Heparin infusion was started during the intervention (50 IU/kg), overlapping with postprocedure oral anticoagulants until the target INR was reached (2–3 according to EASL guidelines^1^). Thereafter, patients were maintained on long-term oral anticoagulation. Anticoagulant was continued for at least 6 months following PTBA in patients with membranous obstruction and for life in patients with segmental obstruction^46^. INR was monitored weekly until the target level was achieved, then once monthly. Management of the underlying hypercoagulable state was done in consultation with hematologists. In addition, symptomatic therapy (low-salt diet, diuretic therapy and/or beta-blockers) was adjusted as needed^8^.

Follow-up data were obtained from the medical records, whenever possible at prespecified intervals (14 days, 1, 3, 6, and 12 months after treatment and then annually or whenever symptoms recurred). Data retrieved included clinical assessment for recurrence of symptoms, laboratory investigations (bilirubin, INR), and imaging (color Doppler US, CT, or MRI). Follow-up ended at the specified timepoint (March 2020) or the timepoint of being lost to follow-up, if the patient underwent TIPS, surgical shunt, or LTx, or by patient death.

## Study endpoints and definitions

The primary endpoints were technical success, clinical success, primary patency duration, and survival. The secondary endpoints were complications and evaluation of factors that could predict long-term patency following recanalization. Technical success was defined as successful recanalization of the target HV with disappearance of intrahepatic collaterals. In patients with stent insertion, technical success also included correct stent positioning, adequate stent expansion (residual stenosis < 30%), and absence of immediate stent migration.

Clinical success was defined as an improvement of BCS-related symptoms (such as ascites, hepatomegaly, hepatic encephalopathy, as well as resolution of portal hypertensive bleeding) and liver chemistries (evidenced by normalization of serum AST/ALT and total bilirubin level,i.e. < 1.5 mg/dL) after technically successful HV recanalization. Complete success was defined as complete elimination of symptoms (diuretics are no longer required). Patients who achieved all except one or two of these parameters including reduction in the dose of diuretics are considered to be partial responders. Clinical failure was considered if there was (a) no improvement, new onset, or recurrence of clinical symptoms, (b) no reduction in the required dose of diuretics, or (c) development or progressive deterioration of liver dysfunction^6,18,36,54^.

Primary patency was defined as the period between the initial angioplasty and re-appearance of outflow obstruction (i.e., recurrence of symptoms which was confirmed by re-occlusion on imaging) that necessitated re-intervention. In patients without re-occlusion, it was the interval until end of follow-up, last follow-up, management by TIPS, surgical shunt, or liver transplant, or patient death. Re-occlusion was suspected on color Doppler US if there was no or retrograde flow, or if there was significant narrowing (> 30%) of the treated segment with formation of intrahepatic collateral vessels and recurrence of BCS-related symptoms. Definite diagnosis of re-occlusion was confirmed by angiography. Secondary patency was defined as the total interval between initial HV angioplasty with the contribution of subsequent recanalization procedures (apart from TIPS) until the last follow-up, end of predefined follow-up period, surgical interventions (surgical shunt or LTx), or death.

Complications were classified according to the guidelines of the Society of Interventional Radiology Standards of Practice Committee^15,54^. Overall survival was defined as the time from start of treatment to last follow-up, end of follow-up, or patient death.

## Statistical analysis

Continuous variables are presented as mean or median and were compared using the independent sample t-test. Categorical variables were compared using the x^2^ test or Fisher exact test. Survival and patency durations were calculated using Kaplan–Meier curves and compared using the log-rank test. Independent predictors of primary patency were calculated using Cox regression analysis. Receiver operator characteristics (ROC) and regression analysis were performed to investigate predictors of hepatic vein patency following angioplasty. A *P*-value < 0.05 was considered statistically significant. All statistical calculations were performed by using Stata/MP version 16.0 (StataCorp, College Station, Texas, USA).

## Supplementary Information

Supplementary Information.

## Data Availability

The datasets used and/or analyzed during the current study available from the corresponding author on reasonable request.
