# Supplementary material for: Percutaneous transluminal angioplasty for symptomatic hepatic vein-type Budd-Chiari syndrome: feasibility and long-term outcomes
Source: Sci Rep. 2022 Aug 18;12:14095. doi: 10.1038/s41598-022-16818-8 (PMC9388522; doi:10.1038/s41598-022-16818-8)
Supplement: Supplementary file 1 — Supplementary Information. [file 41598_2022_16818_MOESM1_ESM.docx]

|  | **Coef.** | **Std. Err.** | **t** | **P>\|t\|** | **[95% Con** |
| --- | --- | --- | --- | --- | --- |
| **Duration of symptoms (months)** | -5.101548 | 3.985785 | -1.28 | 0.220 | -13.59705 |
| **Symptoms** |  |  |  |  |  |
| Liver cirrhosis | 20.87486 | 29.2114 | 0.71 | 0.486 | -41.38776 |
| Ascites | -3.954174 | 19.21348 | -0.21 | 0.840 | -44.90674 |
| Abdominal wall collaterals | 2.573384 | 24.50547 | 0.11 | 0.918 | -49.65878 |
| Varices | -2.262626 | 25.7716 | -0.09 | 0.931 | -57.1935 |
| Hepatic encephalopathy | -5.794481 | 36.87781 | -0.16 | 0.877 | -84.39767 |
| **Laboratory values** |  |  |  |  |  |
| bilirubin | -6.475441 | 17.15312 | -0.38 | 0.711 | -43.03644 |
| AST | .0169457 | .0943653 | 0.18 | 0.860 | -.184189 |
| ALP | -.0852969 | .1124421 | -0.76 | 0.460 | -.3249616 |
| INR | -4.087364 | 21.59961 | -0.19 | 0.852 | -50.12585 |
| **Prognostic indices** |  |  |  |  |  |
| Child-Pugh score | 6.813173 | 6.861396 | 0.99 | 0.336 | -7.811546 |
| Clichy score | 7.78174 | 19.50744 | 0.40 | 0.696 | -33.79739 |
| New Clichy score | 2.350232 | 9.432645 | 0.25 | 0.807 | -17.75497 |
| Rotterdam score | -3.692536 | 25.54248 | -0.14 | 0.887 | -58.13505 |
| Number of occluded veins | 12.66264 | 21.4686 | 0.59 | 0.564 | -33.09661 |
| Nature of obstruction | 2.401512 | 11.42927 | 0.21 | 0.836 | -21.9594 |
| Final TTT | 6.772338 | 12.69389 | 0.53 | 0.601 | -20.28406 |
| **Risk factors** |  |  |  |  |  |
| Anti-phospholipid syndrome | -20.96747 | 38.77094 | -0.54 | 0.597 | -103.6058 |
| Anti-thrombin Ⅲ deficiency | -13.63134 | 44.25275 | -0.31 | 0.762 | -107.9538 |
| FVLM | -20.27151 | 35.03082 | -0.58 | 0.571 | -94.93793 |
| MPD | -31.88449 | 44.21166 | -0.72 | 0.482 | -126.1194 |
| MTHFR | -15.10903 | 42.70476 | -0.35 | 0.728 | -106.1321 |
| No risk factor identified | -24.67196 | 37.6486 | -0.66 | 0.522 | -104.918 |
| Protein C deficiency | -31.78267 | 36.37654 | -0.87 | 0.396 | -109.3174 |
| Protein S deficiency | 9.585528 | 41.20449 | 0.23 | 0.819 | -78.23976 |

**Supplementary table 1:** Regression analysis for predictors of hepatic vein patency following angioplasty.

AST, aspartate aminotransferase; ALP, alkaline phosphatase; INR, international normalized ratio; FVLM, factor V Leiden mutation; MPD, myeloproliferative disorder; MTHFR, methylene tetrahydrofolate reductase.

|  | **Coef.** | **Std. Err.** | **t** | **P>\|t\|** | **[95% Con** |
| --- | --- | --- | --- | --- | --- |
| **Duration of symptoms (months)** | -.3644478 | 2.991351 | -0.12 | 0.905 | -6.740361 |
| **Symptoms** |  |  |  |  |  |
| Liver cirrhosis | -24.82231 | 21.9233 | -1.13 | 0.275 | -71.55071 |
| Ascites | 5.340732 | 14.41981 | 0.37 | 0.716 | -25.39437 |
| Abdominal wall collaterals | -15.15066 | 18.39147 | -0.82 | 0.423 | -54.35115 |
| Varices | 25.08082 | 19.34171 | 1.30 | 0.214 | -16.14507 |
| Hepatic encephalopathy | -18.87079 | 27.67697 | -0.68 | 0.506 | -77.86286 |
| **Laboratory values** |  |  |  |  |  |
| Bilirubin | 1.008228 | 12.8735 | 0.08 | 0.939 | -26.43098 |
| AST | -.074943 | .0708216 | -1.06 | 0.307 | -.2258956 |
| ALP | .0288172 | .0843883 | 0.34 | 0.737 | -.1510523 |
| INR | .6965641 | 16.21061 | 0.04 | 0.966 | -33.85554 |
| **Prognostic indices** |  |  |  |  |  |
| Child-Pugh score | 8.384272 | 5.149511 | 1.63 | 0.124 | -2.591651 |
| Clichy score | -17.7999 | 14.64043 | -1.22 | 0.243 | -49.00523 |
| New Clichy score | 10.69019 | 7.079246 | 1.51 | 0.152 | -4.39887 |
| Rotterdam score | 3.799234 | 19.16976 | 0.20 | 0.846 | -37.06013 |
| Number of occluded veins | -8.90536 | 16.11229 | -0.55 | 0.589 | -43.24789 |
| Nature of obstruction | -3.20973 | 8.577722 | -0.37 | 0.713 | -21.49271 |
| Final TTT | 2.43821 | 9.526829 | 0.26 | 0.801 | -17.86774 |
| **Risk factors** |  |  |  |  |  |
| Anti-phospholipid syndrome | -37.73369 | 29.09778 | -1.30 | 0.214 | -99.75413 |
| Anti-thrombin Ⅲ deficiency | -35.68768 | 33.21191 | -1.07 | 0.300 | -106.4772 |
| FVLM | -34.09239 | 26.2908 | -1.30 | 0.214 | -90.1299 |
| MPD | -2.698704 | 33.18107 | -0.08 | 0.936 | -73.42247 |
| MTHFR | -7.05018 | 32.05013 | -0.22 | 0.829 | -75.36341 |
| No risk factor identified | -32.35899 | 28.25545 | -1.15 | 0.270 | -92.58406 |
| Protein C deficiency | -38.96717 | 27.30077 | -1.43 | 0.174 | -97.15737 |
| Protein S deficiency | -7.822018 | 30.92417 | -0.25 | 0.804 | -73.73532 |

**Supplementary table 2:** Regression analysis for predictors of patient survival.

AST, aspartate aminotransferase; ALP, alkaline phosphatase; INR, international normalized ratio; FVLM, factor V Leiden mutation; MPD, myeloproliferative disorder; MTHFR, methylene tetrahydrofolate reductase.

|  | **Coef.** | **Std. Err.** | **t** | **P>\|t\|** | **[95% Con** |
| --- | --- | --- | --- | --- | --- |
| **Duration of symptoms (months)** | -11.0434 | 10.50954 | -1.05 | 0.404 | -56.2623 |
| **Symptoms** |  |  |  |  |  |
| Liver cirrhosis | 74.33526 | 151.4054 | 0.49 | 0.672 | -577.1098 |
| Ascites | .1291177 | 35.60393 | 0.00 | 0.997 | -153.0622 |
| Abdominal wall collaterals | 60.42999 | 117.222 | 0.52 | 0.658 | -443.9356 |
| Varices | -45.60932 | 110.7113 | -0.41 | 0.720 | -521.9616 |
| Hepatic encephalopathy | 75.01696 | 143.13 | 0.52 | 0.652 | -540.8217 |
| **Laboratory values** |  |  |  |  |  |
| Bilirubin | -.5759127 | 41.56199 | -0.01 | 0.990 | -179.4027 |
| AST | .0610917 | .4686469 | 0.13 | 0.908 | -1.955333 |
| ALP | -.3534243 | .633174 | -0.56 | 0.633 | -3.077752 |
| INR | -23.64068 | 50.35035 | -0.47 | 0.685 | -240.2808 |
| **Prognostic indices** |  |  |  |  |  |
| Child-Pugh score | 4.026894 | 11.82494 | 0.34 | 0.766 | -46.85173 |
| Clichy score | 12.81195 | 37.31925 | 0.34 | 0.764 | -147.7598 |
| New Clichy score | -7.618058 | 19.62446 | -0.39 | 0.735 | -92.05528 |
| Rotterdam score | -10.97005 | 41.12772 | -0.27 | 0.815 | -187.9283 |
| Number of occluded veins | 48.27631 | 62.58511 | 0.77 | 0.521 | -221.0057 |
| Nature of obstruction | 1.482816 | 2.385312 | 0.62 | 0.598 | -8.780351 |
| Final TTT | 12.48825 | 20.41555 | 0.61 | 0.603 | -75.35277 |
| **Risk factors** |  |  |  |  |  |
| Anti-phospholipid syndrome | -33.9964 | 50.3323 | -0.68 | 0.569 | -250.5588 |
| Anti-thrombin Ⅲ deficiency | 4.732982 | 68.69704 | 0.07 | 0.951 | -290.8465 |
| FVLM | -52.63591 | 50.44754 | -1.04 | 0.406 | -269.6942 |
| MPD | -18.14939 | 67.64381 | -0.27 | 0.814 | -309.1972 |
| MTHFR | -21.82357 | 77.85686 | -0.28 | 0.806 | -356.8146 |
| No risk factor identified | -59.46715 | 82.46595 | -0.72 | 0.546 | -414.2895 |
| Protein C deficiency | -49.99558 | 40.95516 | -1.22 | 0.347 | -226.2114 |

**Supplementary table 3:** Regression analysis for predictors of hepatic vein patency following angioplasty in patients with segmental HV occlusion.

AST, aspartate aminotransferase; ALP, alkaline phosphatase; INR, international normalized ratio; FVLM, factor V Leiden mutation; MPD, myeloproliferative disorder; MTHFR, methylene tetrahydrofolate reductase.

|  | **Coef.** | **Std. Err.** | **t** | **P>\|t\|** | **[95% Con** |
| --- | --- | --- | --- | --- | --- |
| **Duration of symptoms (months)** | -9.255691 | 6.054284 | -1.53 | 0.266 | -35.30517 |
| **Symptoms** |  |  |  |  |  |
| Liver cirrhosis | 59.63343 | 87.22091 | 0.68 | 0.565 | -315.6478 |
| Ascites | 5.367305 | 20.51054 | 0.26 | 0.818 | -82.88243 |
| Abdominal wall collaterals | 75.31459 | 67.52868 | 1.12 | 0.381 | -215.2379 |
| Varices | -51.31473 | 63.77802 | -0.80 | 0.506 | -325.7294 |
| Hepatic encephalopathy | 143.2402 | 82.45362 | 1.74 | 0.224 | -211.5291 |
| **Laboratory values** |  |  |  |  |  |
| Bilirubin | -11.15152 | 23.94283 | -0.47 | 0.687 | -114.1692 |
| AST | .0766586 | .2699758 | 0.28 | 0.803 | -1.084954 |
| ALP | -.3256528 | .3647558 | -0.89 | 0.466 | -1.89507 |
| INR | -17.33041 | 29.00559 | -0.60 | 0.611 | -142.1314 |
| **Prognostic indices** |  |  |  |  |  |
| Child-Pugh score | 5.286187 | 6.812056 | 0.78 | 0.519 | -24.02372 |
| Clichy score | -23.9836 | 21.49869 | -1.12 | 0.381 | -116.485 |
| New Clichy score | 4.03443 | 11.30516 | 0.36 | 0.755 | -44.60775 |
| Rotterdam score | -27.04572 | 23.69266 | -1.14 | 0.372 | -128.987 |
| Number of occluded veins | 43.53373 | 36.05373 | 1.21 | 0.351 | -111.5929 |
| Nature of obstruction | -.7007863 | 1.374119 | -0.51 | 0.661 | -6.613142 |
| Final TTT | 6.154315 | 11.76089 | 0.52 | 0.653 | -44.44871 |
| **Risk factors** |  |  |  |  |  |
| Anti-phospholipid syndrome | -63.68543 | 28.99518 | -2.20 | 0.159 | -188.4416 |
| Anti-thrombin Ⅲ deficiency | 13.66862 | 39.57466 | 0.35 | 0.763 | -156.6074 |
| FVLM | -67.47119 | 29.06157 | -2.32 | 0.146 | -192.513 |
| MPD | -19.88681 | 38.96792 | -0.51 | 0.661 | -187.5522 |
| MTHFR | -31.72337 | 44.8514 | -0.71 | 0.553 | -224.7034 |
| No risk factor identified | -95.91086 | 47.50658 | -2.02 | 0.181 | -300.3152 |
| Protein C deficiency | -66.71954 | 23.59325 | -2.83 | 0.106 | -168.2331 |

**Supplementary table 4:** Regression analysis for predictors of survival in patients with segmental HV occlusion.

AST, aspartate aminotransferase; ALP, alkaline phosphatase; INR, international normalized ratio; FVLM, factor V Leiden mutation; MPD, myeloproliferative disorder; MTHFR, methylene tetrahydrofolate reductase.
